# Establishing Cardiovascular Centers on Aging: The Cleveland Clinic Experience

**DOI:** 10.1016/j.jacadv.2025.101987

**Published:** 2025-08-06

**Authors:** Abdulla A. Damluji, Ardeshir Z. Hashmi, Nishok Karthikeyan, Oliver Glen Ancheta, Samir Kapadia, Venu Menon

**Affiliations:** Cardiovascular Center on Aging, Department of Cardiovascular Medicine, The Cleveland Clinic Foundation, Cleveland, Ohio, USA

**Keywords:** aging, cardiovascular diseases, geriatrics, geriatric assessment, multimorbidity, polypharmacy

The world population is aging at an unprecedented rate.^1^ Cardiovascular disease (CVD) remains the leading cause of morbidity and mortality and disproportionately affects the older adult population.^2^ Older adults with CVD are not merely chronologically advanced versions of younger patients. Biological aging goes beyond measuring chronological age or the linear passage of time from birth until old age. It refers to the pathophysiological changes that occur in patients, which in turn lead to declines in function across multiple organ systems. Individually, the trajectory of this decline is variable and influenced by the presence of chronic illnesses, genetic and biological differences, cardiovascular risk, and environmental exposures. When combined with age-mediated functional and structural changes in the cardiovascular system, older patients have distinctive clinical presentations, disease progression, and response to therapeutic drugs and devices. The field of cardiovascular aging reflects all aspects of CVD that affect the older adult population and is focused on providing an optimal approach to diagnostic and therapeutic care delivery for this vulnerable population.

## Why do we need cardiovascular centers on aging?

Most older adults living with CVD live with multiple chronic conditions; more than 65% of Americans^3^ 65 years of age have 2 or more comorbid conditions and more than 80% of those^3^ 75 years of age have multimorbidity.^3^ The concept of multicomplexity arises when multimorbidity is complicated by social factors and geriatric syndromes including polypharmacy, frailty, cognitive impairment, functional decline, and disability. When combined, these factors create complex clinical scenarios that impact diagnostic testing, challenge therapeutic decisions, and undermine medication adherence. Consequently, when older patients with multicomplexity are exposed to therapeutic cardiovascular interventions, their risks/benefit profile is different from those evaluated in randomized clinical trials resulting in a diminished treatment effect and occasionally suboptimal health outcomes.^2^ The current single disease framework for cardiovascular health care delivery is inadequate to address the needs of the older adult population with multicomplexity. Traditional single disease model may inadvertently overlook geriatric syndromes as a fourth pillar of risk.^4^^,^^5^ Thus, a more comprehensive approach moves away from a disease-centric models toward a holistic, patient-centered approach to care, embracing the full spectrum of complexity when managing CVD in older populations.

What further complicates CVD management is the persistent under-enrollment and underrepresentation of older adults in major cardiovascular clinical trials. The exclusion of older, more frail, and multimorbid patients creates substantial gaps in knowledge and leaves clinicians with limited high-quality data to guide decision-making in cardiovascular practice. This lack of evidence presents challenges related to the accuracy in assessing the risks and benefit of diagnostic and therapeutic decisions in the context of biological aging and coexistence of geriatric syndromes. Dedicated centers focused on cardiovascular aging, integrating clinical and translational research, are uniquely positioned to address this gap by generating high-quality evidence tailored to the needs and characteristics of the older adult population. Finally, the economic implications, while not the main focus of such programs, also support the case for specialized cardiovascular centers for aging. The high health care utilization and costs associated with managing multicomplexity in older cardiovascular patients suggest that such programs may be able to reduce utilization including readmissions and shortening hospital length of stay via addressing geriatric complexities such as reducing polypharmacy and managing frailty. Taken together, this can lead to a more efficient use of hospital and clinic resources and provide better value to both the health care system and individual patients.

## Establishing a cardiovascular center on aging: the cleveland clinic experience

The foundation of any specialized center of excellence rests on several principles. These include a clearly articulated vision and goals of the program, a multidisciplinary team of experts, implementation of standardized but patient-centered processes, and commitment to improving quality of care, research operations, and education. These principles should be in alignment with the broader goals of the health care system to align clinicians and staff with goals to achieve higher value through patient-centered care, clinical excellence, and more importantly patient satisfaction. The critical value in establishing the cardiovascular aging center lies in its ability to effectively integrate and reorient cardiovascular services with already established geriatric programs. The example of the evolving Cleveland Clinic Cardiovascular Center on Aging creates a synergistic focus to connecting and coordinating both cardiovascular and geriatric specialized services to serve the unique needs of older adults living with CVD.

The proposed structure of the program consists of 3 arms: clinical services, clinical and translational research, and education and training. The inpatient geriatric medicine consult service is modeled on successful geriatric specialty services. It has garnered recognition as an “Age Friendly Health System-Committed to Care Excellence” by the Institute of Healthcare Improvement and provides expert comanagement for older hospitalized patients with acute cardiovascular conditions. The specialized service focuses on geriatric conditions including delirium, falls, malnutrition, medication optimization, advanced care planning, and functional decline. Preprocedural geriatric cardiology assessment for patients undergoing transcatheter aortic valve replacement, coronary artery bypass graft surgery, high-risk percutaneous coronary intervention, or other mechanical circulatory devices is essential and offers comprehensive risk stratification incorporating frailty, cognitive functioning, nutritional status, and patient goals.^6^ Outpatient geriatric cardiology services provide focused care for complex cardiovascular conditions with routine integration of comprehensive geriatric assessment to identify and manage geriatric syndromes. Cardiac rehabilitation programs are a critical component as a platform tailored to the specific needs of older adults. These programs focus on partnering with patients to prevent frailty, enhance mobility, build endurance, and promote functional independence.

The research arm of the program focuses on the gaps in knowledge pertinent to older adults with CVD. Key areas include pathophysiology of cardiovascular aging, conduct of clinical trials specifically designed for older, frail patients with multimorbidity, evaluating the impact of geriatric syndromes on cardiovascular procedures, and developing validated tools for risk stratification in the older adult populations. Finally, understanding outcomes beyond major cardiovascular adverse cardiovascular (MACE) events in this population remains critical. This is achieved by prioritizing a focus on patient-reported outcomes, quality of life metrics. The program leverages already existing research infrastructure within the heart, vascular, and thoracic institute to build a biobank, patient registry, and prospective cohorts of older patients with CVD to facilitate translational and clinical research.

**Figure 1:**
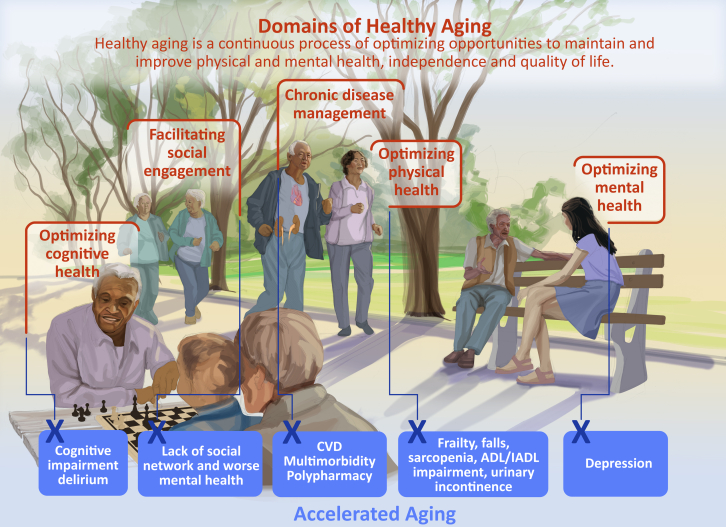
**Domains of Healthy Aging** Healthy Aging is a continuous process of optimizing opportunity to maintain and improve physical and mental health and quality of life. Modified from Schmanske et al. *Eur Heart J*. 2025;ehaf231. ADL = activities of daily living; CVD = cardiovascular disease; IADL = instrumental activities of daily living.

Finally, the educational and training arm of the program builds upon the strengths of both cardiovascular and geriatric medicine fellowship training programs at the institution. A specialized geriatric cardiology training program can provide an advanced training opportunity to integrate geriatric principles in current cardiovascular training. This is in line with the American College of Cardiology initiatives outlined in the Essentials of Cardiovascular Care for Older Adults (ECCOA) (https://www.acc.org/Membership/Sections-and-Councils/Geriatric-Cardiology-Section/About-Us/Section-Initiatives/ECCOA-for-ACC-Chapters-Initiative). Continuing medical education activities and ground rounds enhance integration of both geriatric and cardiology services and provides a platform to disseminate best practices in geriatric cardiology to community-based practices. Finally, development of materials and resources focused on enhancing patients and caregiver education remains critical such as medication management, self-care strategies, and enhancing independence. Such programs may not only improve clinical care but also creates partnerships at the community health level to extend outreach and education on common geriatric syndromes in community settings and provide awareness on how to achieve healthy cardiovascular aging (Figure 1).^1^

## The heart team

An integrated “Heart Team” relies on collaborative interdisciplinary teams providing comprehensive, coordinated patient-centered care.^7^ Ideally, clinicians dual trained in Geriatric and Cardiovascular Medicine are better suited to manage older adults with CVD and multicomplexity. They may provide a unique perspective for managing complex CVD within a geriatric context. Geriatricians provide consultation or comanagement for complex geriatric syndromes, conduct comprehensive geriatric assessments, and advice on age-specific medication management. Advanced practice providers with a focus on geriatrics can play an important role in managing dedicated clinics, ensuring continuity of care, and patient-family follow-up. A clinical coordinator can serve as a central figure for care coordination by helping older adults navigate a complex health system and in facilitating communication between team members, patients, and their families. A geriatric pharmacist is essential for conducting medication reviews and addressing concerns related to polypharmacy, medication adherence, and deprescribing efforts. Other support staff including social workers, physical and occupational therapists, and dietitians can provide further support in psychosocial assessment, functional assessment, and nutritional needs.

## Geriatric principles and cardiovascular care

The systematic integration of core geriatric principles is critical to cardiovascular care. This requires a fundamental shift in how we conceptualize and deliver care to older patients. This involves moving away from a disease-centered approach to a more holistic, patient-centered pathway that prioritizes patient’s preferences and quality of life metrics. The 5M’s of Geriatrics (What Matters, Medication, Mentation, Mobility, and Multicomplexity) provide a framework to integrate and deliver those core principles in cardiovascular care pathways. Routine screening for geriatric syndromes using validated tools remains essential. These include frailty evaluation, cognitive screening, nutritional assessment, polypharmacy review, and deprescribing protocols. Several validated tools exist to measure and manage these syndromes and are discussed in detail elsewhere.^1^ A core principle includes the need for training clinicians on concepts of shared decision-making, particularly for older patients with varying levels of health literacy and cognitive functioning. The Yale originated Patient Priorities Care provides paradigm to capture values, goals, and care preferences of patients with multiple chronic comorbidities.^8^ The use of decision aids helps present information to patients with regard to cardiovascular procedural risk, benefit, and alternative therapies including conservative management. Care coordination and transitioning remain critical between in-patient, out-patient, and community-based settings.

## Measuring success: metrics for CV aging centers

Success for cardiovascular centers of aging extends beyond the traditional, disease-specific metrics such as MACE events.^9^ Such programs’ success is enhanced when measuring patient-centered outcomes, clinical efficacy, process efficiency, quality of care delivery, and research and educational outputs. The ultimate goal is to improve the patient’s experience as they navigate the complex health care system during their acute cardiovascular illness. Key domains and specific metrics for patient-centered outcomes include quality of life (eg, Short Form-36 Health Survey, EuroQol 5 Dimensions), functional status assessment (activities of daily living, instrumental activities of daily living), gait speed, and Short Physical Performance Battery testing. Patient-reported outcomes are also important targets, such as the burden of dyspnea or degree of chest pain. Assessing effectiveness of decision quality, including understanding treatment options and alignment with patient goals and measuring progress toward attainment of goals, all remain important.

Clinical outcomes measures are similar to what is traditionally measured in cardiovascular care pathway such as mortality, MACE, rehospitalization rate, procedural outcomes and complications, but also include tracking the incidence and burden of geriatric syndromes such as delirium, falls, and functional decline. Quality of care metrics are measured via processes including adherence to the proportion of older patients receiving comprehensive geriatric assessments, frailty and cognitive screening, and nutritional assessments. Comprehensive medication assessment and efforts at deprescribing of potentially inappropriate medications are other targets of a successful program. Finally, documented advanced care planning and goals of care discussion are important metrics that should be tracked and documented for all patients. The use of electronic health care systems utilizing “geriatric cardiology sheet” can facilitate the tracking of these metrics over time.^10^

## Future directions and conclusions

The establishment of dedicated cardiovascular centers on aging can present a unique and innovative opportunity to care for the older adult population living with or at risk for CVD. By integrating geriatric principles into comprehensive cardiovascular care, these centers can move beyond a disease-centered approach to management. This approach improves upon traditional MACE metrics and extends to include functional independence, cognitive health, quality of life, and making health care decisions in accordance with patient’s values and preferences. Health systems, academic institutions, and funding agencies must recognize the unique and special needs of the older adult population. A national effort to expand evidence generation to include older and more frail patients with multicomplexity via pragmatic trials is needed with a focus on patient-centered outcomes. Professional societies and patient advocacy groups should champion policies that foster the development of age-friendly cardiovascular care models and address barriers to high-quality care for older patients. There is a challenge in providing optimal care for older CVD patients, but a collaborative effort between geriatricians and cardiovascular clinicians can provide a platform for achieving high-quality care. In light of a rapidly aging population, the establishment of cardiovascular centers on aging can provide an opportunity to achieve the best cardiovascular health and quality of life for older adults.

## Funding support and author disclosures

Dr Damluji has received research funding from the Pepper Scholars Program of the Johns Hopkins University 10.13039/100031147Claude D. Pepper Older Americans Independence Center (OAIC) funded by the 10.13039/100000049NIA P30-AG021334 and has received mentored patient-oriented research career development award from the 10.13039/100000050National Heart, Lung, and Blood Institute K23-HL153771-01. All other authors have reported that they have no relationships relevant to the contents of this paper to disclose.
